# Innate Immune Responses to Herpesvirus Infection

**DOI:** 10.3390/cells10082122

**Published:** 2021-08-18

**Authors:** Christine M. O’Connor, Ganes C. Sen

**Affiliations:** 1 Department of Genomic Medicine, Infection Biology Program, Lerner Research Institute, Cleveland Clinic, Cleveland, OH 44106, USA; 2 Department of Inflammation and Immunity, Infection Biology Program, Lerner Research Institute, Cleveland Clinic, Cleveland, OH 44106, USA

**Keywords:** herpesvirus, HSV, HCMV, KSHV, innate immune response, antiviral host response, intrinsic immunity, innate immunity

## Abstract

Infection of a host cell by an invading viral pathogen triggers a multifaceted antiviral response. One of the most potent defense mechanisms host cells possess is the interferon (IFN) system, which initiates a targeted, coordinated attack against various stages of viral infection. This immediate innate immune response provides the most proximal defense and includes the accumulation of antiviral proteins, such as IFN-stimulated genes (ISGs), as well as a variety of protective cytokines. However, viruses have co-evolved with their hosts, and as such, have devised distinct mechanisms to undermine host innate responses. As large, double-stranded DNA viruses, herpesviruses rely on a multitude of means by which to counter the antiviral attack. Herein, we review the various approaches the human herpesviruses employ as countermeasures to the host innate immune response.

## 1. Introduction

**Figure 1:**
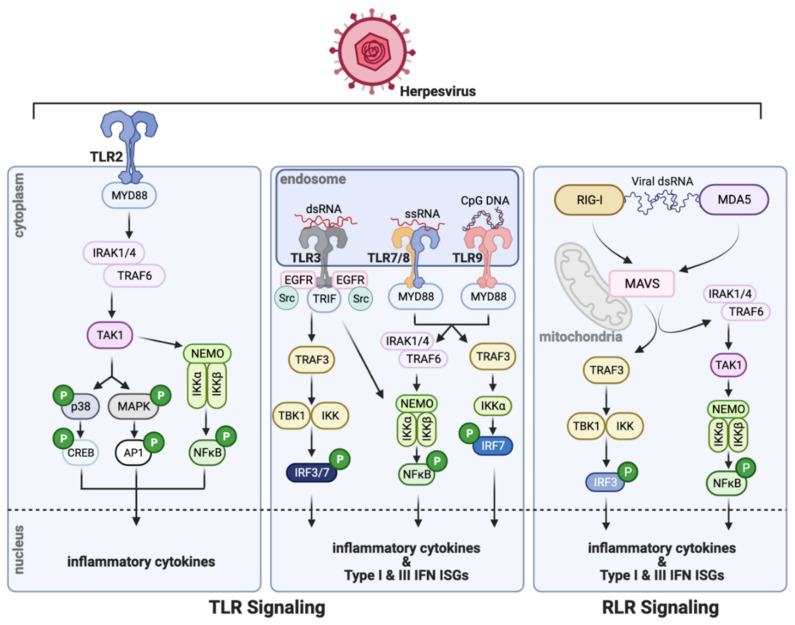
Herpesvirus infection triggers PRR signaling. TLR signaling is induced from the cell membrane (left) or endosomal membrane (middle). TLR2-mediated signaling (left) via MyD88 interaction via TLR2′s cytoplasmic domain, activates transcription factors that in turn initiate inflammatory cytokine and ISG transcription. TLR3, TLR7/8, and TLR9 signaling from endosomes (middle) is activated by dsRNA, ssRNA, or CpG DNA, respectively. Downstream activation of NFĸB, IRF7, or IRF3 leads to the upregulation of inflammatory cytokine and ISG production. The cytoplasmic RLR family of PRRs (right), such as RIG-I, use MAVS as an adaptor for innate signaling, resulting in the activation of inflammatory cytokines and ISGs. The data summarized in this figure is reviewed in ref. [4]. Created with BioRender.com (accessed on 13 August 2021).

Viral infection of mammals triggers multi-tiered antiviral responses that are temporally distinct. Intrinsic protection of the infected cell is provided by immediate induction of many intracellular antiviral proteins, which are encoded by the interferon (IFN)-stimulated genes (ISG) [1,2]. Some ISGs are expressed constitutively, albeit at a low level, and can provide mild antiviral protection. These genes, as their name suggests, are IFN-inducible; however, they can also be induced in virus-infected cells without the involvement of IFN. In addition to ISGs, many cytokines, including type I and type III IFNs, are induced by virus infection (Figure 1). IFNs are secreted to the circulation and act on uninfected cells of the organism to protect them from potential infection, by inducing the ISGs prior to infection. The IFN system represents the most prominent innate immune antiviral defense in mammals. Eventually, T cell and B cell-mediated adaptive immunity, directed toward the specific virus, takes over the protective functions. Viruses have evolved to counteract the host’s immune defense through various evasion strategies; large DNA viruses, such as herpesviruses, employ a plethora of viral proteins for this purpose [3].

**Figure 2:**
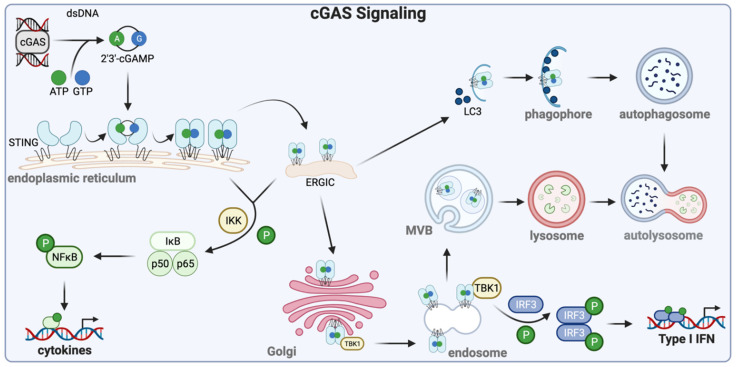
cGAS-STING signaling induces a potent innate immune response. DNA in the cytosol activates cGAS, inducing the formation of a cGAS-DNA dimer. This dimeric complex uses ATP and GTP to then synthesize 2′,3′-cGAMP, which then binds and activates STING via conformational changes in the endoplasmic reticulum. Coat protein complex II (COP II) transports STING as its cargo to ERGIC Activated STING is then translocated to the Golgi or targeted for autophagy via LC3. From the Golgi, activated STING can shuttle to endosomes, where it either undergoes lysosomal degradation via multivesicular bodies (MVB) or recruits TBK1, resulting in IRF3 phosphorylation. Active IRF3 then dimerizes, translocates to the nucleus, and drives the type I interferon response. Alternatively, activated STING phosphorylates IKK, ultimately leading to NFĸB activation. Nuclear translocation of NFĸB induces cytokine production. Created with BioRender.com (accessed on 13 August 2021).

There are specific cellular proteins, called pattern recognition receptors (PRR), that recognize components of the infecting viruses and orchestrate appropriate cellular responses [5]. A prominent group of PRRs is the Toll-like receptors (TLR; Figure 1), which are transmembrane proteins spanning the plasma membrane or the endosomal membrane [6]. Their extracellular domains or the endosomal luminal domains recognize the viral ligands whereas the cytoplasmic domains bind to the proteins, MyD88 (myeloid differentiation primary response protein 88) or TRIF (TIR-domain-containing adapter-inducing interferon-β), and assemble the proteins of the signaling complexes. The intracellular endosomal TLRs, the major players in viral recognition, are activated by various nucleic acids that need to be endocytosed; double-stranded RNA is the ligand for TLR3, TLR7, and TLR8 bind single-stranded RNA whereas TLR9 binds DNA. Cytoplasmic dsRNA or 5′ppp RNA is recognized by retinoic acid-inducible gene-I (RIG-I), the founding member of the RIG-I-like receptor (RLR) family of cytoplasmic PRRs (Figure 1), which uses the mitochondrial membrane-bound protein, mitochondrial antiviral-signaling protein (MAVS), as the adaptor for signaling [7]. Viral or cellular cytoplasmic DNA is recognized by several cytoplasmic or nuclear proteins that can initiate an innate immune response. The most prominent protein in this group is cyclic GMP-AMP synthase (cGAS), an enzyme that uses cytoplasmic DNA as the co-factor to synthesize the 2′-3′ cyclic dinucleotide, cGAMP (Figure 2) [8]. This and other cyclic dinucleotides, produced by intracellular bacteria, bind to the ER-bound protein, stimulator of interferon genes (STING)/mediator of IRF3 activation (MITA), which serves as the platform for signaling complex assembly. In addition to cGAS, interferon-gamma inducible protein 16 (IFI16), DEAD-box helicase 41 (DDX41), and DNA-dependent activator of IFN-regulatory factors (DAI; a.k.a Z-DNA-binding protein 1, ZBP1) are purported as cytoplasmic DNA sensors, although their exact modes of action remain unclear.

Transcriptional signaling by all antiviral PRRs is elicited by biochemical pathways that are quite similar, although the mechanistic details vary [9]. Ligand-binding changes the conformation of the PRR and often leads to its dimerization, allowing recruitment of protein kinases, that phosphorylate Ser/Thr or Tyr residues of the receptor, and E3 ligases that ubiquitinate Lys residues. These enzymes also modify other adaptors and signaling proteins that constitute the signaling complex. The cascade of post-translational modifications extends to transcription factors recruited by the signaling complex; these factors physically transmit the signal to the nucleus. The two most relevant transcription factors for the IFN system are interferon regulatory factor 3 (IRF3) and nuclear factor-ĸB (NFĸB), which together drive transcription of the IFN-β mRNA. NFĸB is activated by its release from its inhibitor IĸB, as a consequence of phosphorylation of the inhibitor by IKKs present in the signaling complex. NFĸB moves to the nucleus, binds to the ĸB sequences present in the regulatory regions of many cytokine genes, and drives their transcription. Cytoplasmic IRF3 is activated by phosphorylation of specific Ser residues by the protein kinase, TANK binding kinase 1 (TBK1); activated IRF3 dimerizes, translocates to the nucleus, and binds to the interferon-sensitive response element/IRF response element (ISRE/IRE) sequences of the target genes to induce their transcription. IRF3 belongs to the large family of IRFs proteins, the gatekeepers of the IFN system [10]. All of them can bind to the ISRE sequences in gene promoters but may have different functional properties. Some, such as IRF3 and IRF7, promote transcription themselves, whereas others, such as IRF9, lack activation domains and require help from associated transcription factors. Some IRFs, such as IRF8, can even repress gene transcription. Some are ubiquitously expressed whereas the expression of others is cell type-specific. Several herpesviruses encode viral IRFs that interfere with the cellular IFN system [11].

Virally induced IFNs are secreted and bind to the cell-surface receptor, interferon-α/β receptor (IFNAR), to elicit their actions. Cells of almost all lineages can produce type I IFNs and respond to them by inducing the transcription of hundreds of ISGs. IFNAR-bound Janus kinases (JAK) activate the transcription factors, signal transducer and activator of transcription 1 and 2 (STAT1 and STAT2), by phosphorylating their specific Tyr residues; activated STAT1 and STAT2 bind to IRF9 and the trimeric complex, ISG factor 3 (ISGF3), moves to the nucleus to bind to ISRE sequences of the ISGs and drive their transcription. The proteins encoded by many ISGs have antiviral effects. Their actions are often specific for different families of viruses and can be cell type-specific as well. They orchestrate multi-pronged attacks by inhibiting different steps of virus replication and the overall antiviral effects are cumulative [12].

### The Human Herpesviruses

In this review, we focus on the human herpesviruses. This double-stranded DNA virus family includes nine, structurally similar viruses that can be further divided into three subfamilies. Another distinct, common trait of the *Herpesviridae* is their life-long infection with their hosts. Following initial infection, herpesviruses establish a latent infection, resulting in the carriage of the viral genome in the absence of viral replication. Arguably, herpesviruses persist in this latent phase for the majority of the time, however sporadic reactivation events allow for viral transmission within and among individuals. The lytic replication stage that follows viral reactivation leads to various diseases, ranging from cold sores and rashes to retinitis and encephalitis, and in some cases, cancer. As viruses that harbor large genomes, the herpesviruses utilize their genomic real estate wisely, having co-evolved with their host to encode their own factors, which enable them to counter the robust host immune response. The human herpesviruses have devised mechanisms to subvert the innate immune response, as well as usurp the pathways involved to their own advantage. Herein, we review the current literature that highlights human herpesvirus inhibition and exploitation of the innate immune responses, using herpes simplex virus (HSV), human cytomegalovirus (HCMV), and Kaposi’s sarcoma-associated herpesvirus (KSHV) as examples.

## 2. HSV

HSV-1, a prototype human alphaherpesvirus, is prevalent worldwide [13]. Oral mucosal epithelial cells are the primary targets of infection; following replication in the epithelia, the virus enters the nervous system through the sensory axons and retrograde transport to cell bodies to establish life-long latent infections primarily in trigeminal ganglia or dorsal root ganglia [14]. In neurons, viral latency is maintained by the host innate immune system. As discussed below, HSV-1 infection results in upregulation of various innate responses, which the virus counteracts through a number of its own proteins (Table 1).

### 2.1. PRR-Mediated Responses to HSV

Cellular antiviral response to HSV-1 infection is initiated by many PRRs that recognize viral proteins and nucleic acids; however, it is not clear whether only a few of them are important in controlling viral pathogenesis or whether different cell types use the PRRs differentially [15]. Most of the supporting evidence, regarding the identity of the specific PRRs that respond to HSV-1 infection, comes from genetically modified cells or mice; but familial susceptibility to viral encephalitis in humans has also provided important clues. The cell surface receptor TLR2 is activated by infecting virions and triggers inflammatory cytokine and chemokine production [16], which contributes to the disease; consequently, TLR2-/- mice are less susceptible to HSV-1-induced death [17], highlighting the importance of this particular PRR. In contrast, virally induced type I IFN is critically important for protecting the infected mice. Among the endosomal nucleic acid recognizing TLRs, TLR3 is the most relevant one for providing protection by inducing IFN synthesis upon HSV-1 infection [18]. Strong evidence for a protective role of TLR3 in HSV-1-mediated encephalitis has come from studies in patients who have inherited defects in the TLR3 gene or genes encoding proteins that mediate TLR3 signaling [19]. Interestingly, the cytoplasmic RIG-I-like receptors have been implicated in the cellular response to HSV-1 infection as well [20,21]. Cytoplasmic DNA binding by cGAS/STING plays an important role in sensing and inhibiting HSV-1 infection [22,23,24]. Another DNA-binding protein, IFI16, can mediate IFN induction by HSV-1; however, it is unclear whether it encounters viral DNA in the nucleus or the cytoplasm [25,26], necessitating additional studies aimed at unraveling the specific biological mechanisms. In the second phase of the IFN response, virally induced secreted IFN binds to cell surface receptors and induces transcription of many ISGs. Although IFN pre-treatment of cells inhibits HSV-1 replication, the identity of the ISGs that mediate this effect remains unclear. In many studies, viral mutants that lack IFN antagonists were used; but the putative antiviral ISGs could inhibit only the mutant, not the wild-type virus. Two ISGs, ISG15 and mycovirus resistance B (MxB), are the prime candidates for inhibiting wild-type HSV-1 [27,28]. Identifying the roles of specific ISGs during HSV-1 infection will undoubtedly bolster our understanding of how these host proteins regulate the infection response. 

### 2.2. HSV Innate Immune Evasion Strategies

The importance of the IFN system in controlling virus replication is reflected in the multi-pronged evasion strategies that HSV-1 has developed to successfully infect the host and establish latency. Different viral proteins can block IFN induction, IFN signaling, ISG transcription, or ISG functions. Many such proteins are packaged in the teguments of infecting virions and delivered to the cytoplasm of the infected cells right from the start of infection, whereas others are newly synthesized, early after the infection begins.

HSV-1 ICP0, a ubiquitin E3 ligase, can inhibit TLR2-mediated inflammatory response by promoting its degradation [29]. It also reduces the levels of MyD88 and MAL (megakaryoblastic leukemia 1; a.k.a. TIRAP, toll/Interleukin-1 receptor domain-containing adaptor protein), two TLR adaptor proteins [30]. TLR3 expression, on the other hand, is inhibited by the viral tegument protein kinase, US3 [31], which also inhibits polyubiquitination of TNF receptor-associated factor (TRAF) 6, another adaptor of TLR signaling [32]. Signaling by the cytoplasmic RLR pathway, which leads to IFN induction, is blocked by several HSV-1 proteins. US11, an RNA-binding tegument protein, binds to both RIG-I and MDA5 and inhibits their interaction with MAVS, the common adaptor required for triggering signaling [33]. TRAF3 is an essential component of this pathway and it requires K63-linked polyubiquitination for its signaling action. HSV-1 UL36, a ubiquitin-specific protease, and the largest tegument protein degrades TRAF3 and prevents its recruitment of TBK1, the protein kinase that phosphorylates IRF3 [34]. Incorporating such a protein into the viral tegument is a clever strategy HSV-1 devised to ensure immediate action against this potent antiviral response.

Cytoplasmic DNA sensing plays an important role in mounting IFN response to HSV-1 infection. The cGAS/STING pathway, which is used by the virus to induce IFN, is blocked by several viral proteins. UL37 deamidates cGAS to block its enzymatic activity [35]. ICP27 targets TBK1-mediated STING signaling [36] and UL41 binds to STING and blocks its translocation from the ER to other membrane compartments, a process that is necessary for signaling [37]. Recently, we have uncovered a novel pathway by which HSV-1 evades STING signaling: it induces a microRNA (miRNA) that inhibits STING synthesis by binding to its mRNA [38]. Another relevant DNA-sensing protein is IFI16, and ICP0, possibly in conjunction with other viral proteins, promotes its degradation [39], suggesting HSV-1 encodes multiple factors to ensure the success of such viral countermeasures.

TBK1, which is used by all IFN-inducing pathways to activate IRF3, can be sequestered by binding to the viral protein, ICP34.5 [40]. The tegument protein UL46 inhibits TBK1 as well [41], which again highlights the multifaceted approach the virus elicits to counter the host innate response. IRF3 itself is hyperphosphorylated by the viral protein kinase US3, which prevents its activation by TBK1 [34]. VP16, on the other hand, blocks IRF3-CBP interaction [42]. US3 also hyperphosphorylates and inactivates p65, a subunit of the transcription factor NFĸB, which is required for IFNβ induction [43].

In addition to blocking IFN induction by multiple mechanisms, HSV-1 inhibits the functions of several ISGs. The dsRNA-activated kinase, protein kinase R (PKR), is inhibited by US11, which binds to both PKR and dsRNA and prevents their direct interaction [44]. Similarly, US11 blocks PKR activation by its protein activator, PACT [45], and also abrogates IFN-induced 2′,5′-oligoadenylate synthetase (OAS) activation by dsRNA [46]. The tegument protein, UL41, blocks the antiviral activity of IFIT3 (interferon-inducible protein with tetratricopeptide repeats 3) [47]. UL41, which promotes host mRNA degradation, can target the mRNAs of both viperin (virus inhibitory protein, endoplasmic reticulum-associated IFN-inducible) and ZAP, two other ISGs [48,49].

Finally, tetherin (a.k.a Bst-2 or CD317), which is an IFN-inducible gene, restricts HSV-1 particle release [50,51] by trapping mature virions on the cell surface, leading to their lysosomal degradation. As a countermeasure, HSV-1 *UL41* encodes the virion host shutoff protein (Vhs), which allows the virus to subvert tetherin trapping [51] along with glycoprotein M (gM) [50]. While this IFN-induced restriction factor functions against several viruses (reviewed in [52]) in addition to HSV-1, its antiviral actions are not conserved across the herpesviruses. While tetherin inhibits KSHV virion release and is antagonized by KSHV-encoded K5 [53,54], this is not the case for HCMV. In fact, tetherin enhances HCMV entry, thus bolstering, as opposed to restricting viral infection [55]. Such differences likely reflect subtle variances between the viruses themselves, the cells they infect, and the specific, host antiviral responses.

## 3. HCMV

HCMV, arguably the more studied of the human betaherpesviruses, is a ubiquitous pathogen, and ~60–80% of adults over 40 years of age are latent carriers. Reactivation of HCMV usually requires a significant weakening of the immune system, such as during immunodepletion in the case of organ transplant or immunosuppression, as is frequently observed in AIDS patients. Additionally, congenital CMV infection is the most common cause of congenital birth defects in the US and can lead to neurodevelopmental defects and hearing loss. Like the HSV-1, HCMV has devised a myriad of mechanisms to subvert the host innate response (Table 2), although recent evidence also suggests this betaherpesvirus coopts this host response to its own advantage.

### 3.1. HCMV Tegument Proteins Confer Immediate Action

Like all herpesviruses, the HCMV virion’s tegument proteins are comprised of both host and viral factors, which can immediately act upon entry into a newly infected cell. HCMV tegument proteins, pp65 and pp71, encoded by *UL83* and *UL82*, respectively have evolved functions to interfere with several innate host responses.

The cGAS-STING-IRF3 signaling pathway is rapidly induced following HCMV lytic infection of endothelial [56] and fibroblast [57] cells, which is not only required for IFN-I induction but necessary to suppress viral replication [56]. Furthermore, the PYHIN family protein, IFI16, detects viral DNA upon infection to further restrict viral replication (reviewed in [58]). While IFI16 senses both cytoplasmic and nuclear viral DNA [59], its ability to sense herpesviral DNA, including that of HCMV, is likely restricted to the nucleus, as the stable icosahedral viral capsid encloses the viral genome, protecting it until delivery into the nucleus. This notion is supported by recent work in IFI16-mediated transcriptional silencing in the context of KSHV [60]. HCMV *UL83*-encoded pp65 is the most abundant tegument protein [61], and its incorporation into the mature particle is certainly not by mistake, as it can immediately function upon infection (reviewed in [62]). Indeed, Cristea et al. showed pp65 interacts with IFI16 [57,63] to block IFI16 oligomerization by targeting its pyhin domain [57]. Consequently, this blocks the activation of STING and subsequent host antiviral responses [57]. More recently, Biolatti and colleagues showed pp65 specifically binds and inactivates cGAS, thereby preventing its interaction with STING and restricting IFNβ expression in lytically infected fibroblasts [64]. While the net effect is similar, it is important to note that Biolatti et al. reported pp65′s modulation of this pathway was independent of STING [64]. Collectively, these studies reveal the complexity of pp65′s involvement in regulating the cGAS-STING antiviral response and highlights the need to better understand this regulation in the context of HCMV infection.

While pp65 may be the most abundant tegument protein in the HCMV virion, it is not the only one that has devised mechanisms to thwart the host antiviral measures. *UL94* encodes a late protein that aids in secondary envelopment [65] and is incorporated into the tegument layer [66]. pUL94 interacts with STING, thereby disrupting both its dimerization and translocation. In turn, this prevents TBK1 recruitment, effectively abrogating STING signaling and downstream IFNβ expression [67]. Additionally, *UL82*-encoded pp71 negatively regulates cGAS-STING signaling [68]. As with pp65′s interaction with cGAS described above, pp71 interacts with STING, which prevents the subsequent activation of this antiviral signaling axis. pp71 has evolved two strategies by which to target STING. The pp71-STING interaction results in the inability of STING translocation from the ER via the Golgi to perinuclear microsomes. Not only is trafficking disrupted, but TBK1 and IRF3 recruitment to the STING complex is abrogated [68]. During the innate response, TBK1-mediated phosphorylation of IRF3 following their recruitment to the complex would otherwise initiate the expression of downstream antiviral genes [69,70,71,72,73,74], which is subverted by pp71 during HCMV infection [68]. Unsurprisingly, the host cell has devised yet another countermeasure to offset pp71′s evasion of cGAS-STING signaling. Nukui and colleagues identified a post-translational modification, called protein S-nitrosylation, which specifically modifies pp71 at cysteines 34, 94, and 218. While mutations of each of these cysteines to serines did not impact incorporation of pp71 into the viral particle or viral growth, the C218S mutant increased pp71′s interaction with STING and IFNβ production [75]. Thus, pp71 protein S-nitrosylation decreases this tegument protein’s interaction with STING, leading to upregulation of antiviral cytokines.

That HCMV has devised several strategies to counter the robust cGAS-STING antiviral pathway is not surprising. In addition to targeting the main players in this cascade, several groups have recently shown that ISGylation is activated following HCMV infection [76,77,78]. ISGylation is induced by cGAS-STING signaling and occurs when ISG15 conjugates proteins in a similar process of protein ubiquitylation. A recently identified ISG15 target protein is the HCMV tegument protein, pUL26. Not only does this lead to ISGylation of pUL26 itself, but also functions to suppress the accumulation of other ISGylated proteins [76,77]. While not a tegument protein, the *UL123*-encoded immediate-early protein, IE1, significantly attenuates *ISG15* expression [77]. IE1 is one of the first proteins expressed by HCMV following infection, thus by targeting ISG15 by a virion-associated protein as well as IE1, HCMV ensures a rapid counterattack to host-mediated ISG15 upregulation as an antiviral measure. These rapid responses from both the cell and virus highlight the exquisite and complicated interplay between the pathogen and host. Similarly, pUL50, a transmembrane protein and a component of the nuclear egress complex, also inhibits global ISGylation [78]. Thus, while the tegument proteins initiate this antagonism, it is clear HCMV has devised strategies to continually subvert this innate immune response throughout its lifecycle.

The HCMV tegument protein, pUL23 [79], also targets the host innate immune response [80]. Subsequent to cGAS-STING activation, host cells upregulate IFNγ and its downstream signaling pathways to induce a potent antiviral response. Most notable, JAK-STAT signaling is modulated, which in turn leads to the transcription of ISGs. Unsurprisingly, HCMV has devised several strategies to target STAT signaling at immediate-early times of infection through both IE1 and IE2 [81,82,83,84,85]. More recent findings suggest HCMV targets STAT signaling even earlier in the infection process. N-myc interactor (NMI) binds to all STATS, except STAT2, [86,87,88,89] and bolsters STAT signaling by recruiting other transcriptional regulators (e.g., CBP/p300), thus enhancing the IFNγ response [89]. Feng and colleagues showed pUL23 interacts with NMI following HCMV infection of human glioblastoma U251 cells, sequestering NMI to the cytoplasm. Furthermore, this also resulted in cytoplasmic localization of STAT1, thereby leading to a significant decrease in ISG transcription [80]. While this was the first report of a herpesvirus protein interacting with NMI, it is not unreasonable to speculate other herpesvirus tegument-associated proteins may function similarly. Future work aimed at identifying other potential herpesviral-encoded NMI interacting proteins will reveal whether this is a conserved mechanism among the herpesvirus family to evade the host antiviral response.

### 3.2. Other HCMV-Encoded Proteins Involved in Innate Immune Evasion

As alluded to above, in addition to delivering virion-associated proteins that can target the host innate responses, HCMV also encodes several proteins throughout its lifecycle to continually counter the antiviral response. The immediate-early protein IE2 (encoded by *UL122*) binds NFĸB following infection, thus preventing its association with the IFNβ promoter, leading to its suppression [90]. However, more recent work revealed that IE2 targets STING for degradation, furthering *IFNB* transcription [91], again demonstrating HCMV has devised a multifaceted approach to counter cGAS-STING’s antiviral effects.

HCMV also interferes with RIG-I/MAVS, in parallel to cGAS-STING signaling. pUL37x1, or viral mitochondria-localized inhibitor of apoptosis (vMIA), is expressed at immediate-early times of infection, at which point it acts to suppress MAVS signaling [92,93]. Independent of its role in apoptosis (reviewed in [94]), vMIA also counters RIG-I/MAVS by degrading RIG-I following infection of primary human foreskin fibroblasts [95]. It is quite likely other HCMV-encoded proteins target RIG-I/MAVS signaling, and undoubtedly more work in this area will lead to a better understanding of how HCMV manipulates this arm of the innate immune response.

It is also becoming apparent that HCMV requires sustained evasion of the innate response throughout its lytic life cycle. As infection progresses, the viral early proteins are expressed, and at least one of these, encoded by *US9*, continues to manipulate type I IFN signaling. Like other HCMV proteins, pUS9 also interacts with STING to inhibit its dimerization and subsequent activation of IRF3. Additionally, and in a mechanism apparently distinct from its interaction with STING, pUS9 targets MAVS, which prevents its ability to recruit TBK1 and IRF3 [96]. This is a clever, multifaceted approach to ensure IRF3 is not activated downstream, which likely allows HCMV to continue its infection cycle while dampening an important host antiviral response.

In further support of HCMV’s continued manipulation of the host immune response is recent work revealing the involvement of pUL31 and pUL42 in this process [97,98]. Similar to other HCMV proteins discussed herein, pUL42 interacts with cGAS, which prevents cGAS’s ability to bind DNA, as well as oligomerize or activate cGAMP production. Additionally, pUL42 interacts with STING, retaining it in the ER and preventing its activation. Deletion of the UL42 open reading frame (ORF) from the viral genome fails to replicate to wild type levels [97], suggesting pUL42′s functions are critical to impeding the host-mediated viral restriction. HCMV sustains its attack on cGAS-STING signaling through late times of infection through the expression of pUL31, a protein encoded a ‘true late’ times of infection [99]. Huang and colleagues showed expression of pUL31 results in its direct interaction with cGAS, leading to dissociation of cGAS from DNA and in turn attenuation of cGAMP production. Conversely, infection of cells with a mutant HCMV lacking pUL31 expression resulted in a significant upregulation of type I IFN, as well as downstream antiviral genes, including *IFNB1*, *RANTES*, and *IL6*. Supporting pUL31′s role in countering the antiviral response, lytic infection of fibroblasts with the UL31-deletion mutant results in attenuated viral growth, when compared to wild type infection; a phenotype that is reversed by infection of cells devoid of cGAS [98].

As discussed above, ISG transcription is a key downstream component of innate immune activation. In addition to ISG15, other examples include IFIT proteins, interferon-inducible transmembrane (IFITM) proteins, mycovirus resistance A (MxA), and MxB, as well as viperin. At present, the role of IFITM proteins in the context of HCMV infection remains unclear. Traditionally, IFITMs mainly function to abrogate viral entry by disrupting membrane fusion between the virus and the host cell (reviewed in [89]). Contrary to what one might predict, overexpression of IFITM1, IFITM2, and IFITM3 actually lead to a slight increase in HCMV infection, as opposed to inhibiting it, in both HeLa cells [100] and primary MRC-5 embryonic lung fibroblasts [101]. Consistent with this, shRNA-mediated knockdown of these same three IFITMs resulted in attenuated viral growth, when compared to virus grown in control cells [101]. Furthermore, IFITM expression during late phases of lytic infection was required for the formation of the viral assembly compartment [101], a perinuclear structure, that while still poorly understood, is a site at which many virion-associated proteins localize during the assembly process [102,103,104,105,106,107,108,109,110,111]. Despite these data showing the requirement for IFITMs in the assembly process, Xie and colleagues also found that at similar times post-infection, HCMV actually downregulated IFITM expression [101]. Why, then, would HCMV attenuate proteins that it requires for its successful assembly and egress? It is likely there is a fine-tuned balance HCMV requires for IFITM expression, yet this still remains unclear and further highlights the complexity of these proteins in the infection process.

The ISGs, MxA, and MxB, are each upregulated in response to HCMV infection [27,112,113]. These dynamin-like GTPases have been well-described antiviral proteins for many RNA viruses, as well as some DNA viruses (reviewed in [114]). How these antiviral proteins attempt to undermine HCMV infection is not fully elucidated, but for MxB, it appears this host-encoded ISG restricts the replication of many herpesviruses, including HSV-1, HSV-2, KSHV, and HCMV [27,113]. It is reasonable to hypothesize a conserved herpesvirus-encoded protein likely counters the actions of MxB, but such a protein has not yet been identified. HCMV also targets the ISG, OAS1 [115], which along with RNase L, represents a critical conduit in the antiviral immune response of many viral pathogens (reviewed in [116]). HCMV immediate-early proteins IRS1 and TRS1 both target OAS/RNase L signaling [117,118], while HCMV pORF94 inhibits both the expression and functionality of OAS1 [115]. It is important to note that pORF94 is expressed during both the lytic and latent phases of infection. It will therefore be interesting to see how pORF94, along with other latency-associated proteins like US28 and UL138, regulate innate immune responses during the latent phase of infection, as well as when the virus reactivates in response to proper stimuli. Work during these phases of infection are in their infancy, but perhaps we can garner some clues from the other herpesviruses, including KSHV, discussed below.

## 4. KSHV

Kaposi’s sarcoma-associated herpesvirus (KSHV; human herpesvirus-8, HHV-8) is a member of the gammaherpesvirus subfamily of herpesviruses. KSHV is the etiologic agent of KS, the most common cancer in people living with HIV (PLHIV), as well as plasmablastic multicentric Castleman’s disease (MCD) and primary effusion lymphoma (PEL) (reviewed in [119]). While most KSHV infections remain asymptomatic, the virus is transmitted via body fluids, where it can go on to infect a variety of cell types. As with HCMV, immune-naïve populations, as well as immunodeficient populations (e.g., PLHIV, aging populations) render KSHV-infected individuals susceptible to viral reactivation and KS development. In response to a fighting immune system, it is no surprise that KSHV has devised its own strategies to successfully evade the host response (Table 3).

### 4.1. Virion-Associated Proteins—Tegument Antagonism of Innate Responses

KSHV targets RIG-I/MAVS signaling not only during initial infection but also during viral reactivation. Indeed, RIG-I or MAVS shRNA knockdown cells infected with KSHV yield an increase in viral transcripts, coupled with a decrease in IFNβ levels [120]. Perhaps to counter the RIG-I/MAVS response, KSHV *ORF64* encodes a tegument-associated protein, which functions as a deubiquitinase (DUB) and is important for efficient lytic replication [121]. However, as a tegument protein, ORF64 can function immediately upon viral infection. Ubiquitination of RIG-I by TRIM25 triggers the IFN pathway, and furthermore, this modification is required for RIG-I’s subsequent interaction with MAVS [122]. In the face of KSHV infection, however, ORF64 interferes with the ubiquitination of RIG-I, thereby attenuating downstream IFN signaling [123].

KSHV *ORF45* encodes an immediate early gene [124], whose protein product is incorporated into the tegument of mature viral particles [125]. Following infection, IRF3 and IRF7 are phosphorylated by the virus-activated kinase components, IKKε and TBK1 [126]. However, ORF45 attenuates IRF7’s activity and prevents its nuclear accumulation, thereby dampening the type I interferon response [127,128,129]. ORF45 does not directly target IRF7 phosphorylation, but rather acts as a competitive substrate for the two cellular kinases, as each effectively phosphorylates ORF45 more efficiently than IRF7. Importantly, this ORF45-mediated phenotype is specific for IRF7, as IRF3 remains unaffected [130]. It is not unreasonable to speculate KSHV may have evolved a separate, yet parallel mechanism, to target IRF3, though this remains to be elucidated. Indeed, recent work by Meng and Gao revealed exportin 1 (XPO1; a.k.a CRM1) inhibition results in increased interferon signaling, preceded by bolstered activation of both IRF3 and TBK1, culminating in a significant decrease in lytic replication in endothelial cells [131]. XPO1 is usurped by many viruses that exploit this cellular protein for viral replication and transport of both viral and cellular proteins (reviewed in [132]), and KSHV is no exception, as XPO1 regulates ORF45’s nuclear export [133]. However, XPO1 inhibition, which would restrict ORF45′s ability to shuttle to the cytoplasm, is likely not responsible for the attenuated viral replication observed by Meng and Gao [131], as retention of ORF45 in the nucleus following mutation of the nuclear export sequence allows for viral growth similar to that of wild type [133]. It is important to note that cell type may impact these findings. The earlier work detailing ORF45 nuclear shuttling was performed in 293T cells [133], while the more recent experiments utilized primary human umbilical vein endothelial cells (HUVEC) [131]. Thus, future studies aimed at understanding the interplay between these proteins will undoubtedly shed light on the potential intersection of KSHV-mediated IRF3 and IRF7 regulation.

The tegument protein, ORF63, shows significant homology to the cellular NLR (nucleotide-binding and oligomerization, leucine-rich repeat) protein, NLR family pyrin domain containing 1 (NLRP1) [134]. When activated by PAMPs, NLRs form inflammasomes, which allow for the proteolytic processing of inflammatory cytokines. Upon infection, ORF63 inhibits NLRP1-dependent processes, including the activation of caspase-1 and degradation of IL-1β and IL-18 [134]. As one might hypothesize, NLRP1 inhibition led to not only efficient reactivation from latency but was also necessary for de novo virion production [134]. These findings suggest KSHV ORF63 acts as an NLR decoy, allowing the virus to successfully reactivate and replicate in host cells. That KSHV has evolved to encode a protein that it incorporates in the virion, as well as functions during reactivation and lytic replication implicates NLR regulation as a key point of regulation. Whether this is shared among other gammaherpesviruses, or more generally, the other subfamilies of herpesviruses remains unknown.

In parallel, the gammaherpesvirus-specific tegument protein, ORF52, binds both cGAS and DNA to prevent cGAS signaling to STING and TBK1, thus inhibiting downstream IFNβ production [135]. In addition to targeting this pathway during lytic infection, like RIG-I/MAVS signaling, KSHV targets the cGAS-STING cascade during viral reactivation. Indeed, siRNA-mediated knockdown of either cGAS or STING resulted in bolstered KSHV reactivation from latency [136]. Furthermore, cytoplasmic isoforms of the latency-associated nuclear antigen (LANA), interact with cGAS, thereby preventing the downstream activation of TBK1-IRF3, likely aiding in KSHV’s ability to successfully reactivate [137]. Since cGAS-STING signaling is such a potent pathway in the innate response, it should be of no surprise that KSHV encodes additional proteins to target this pathway, discussed in detail below.

### 4.2. Virion-Associated Proteins—Viral IRFs

Distinct to KSHV are the viral IRFs (vIRFs), encoded by K9 (vIRF1), K11 and K11.1 (vIRF2), K10.5 and K10.6 (vIRF3), and K10 (vIRF4). While these vIRFs are multifunctional (reviewed in [138]), Jacobs and colleagues showed at least three of these, vIRFs 1, 2, and 3, inhibit TLR3-mediated type I interferon signaling via discrete mechanisms [139]. Additionally, vIRF4 interacts with IRF7 to prevent its dimerization and downstream production of IFNα [140], while also directly targeting cellular IRF4 [141,142]. These vIRFs also have evolved to target cGAS-STING signaling. Similar to the other two herpesvirus subfamilies, the cGAS-STING pathway targets KSHV upon infection. KSHV infection of endothelial cells results in IFNβ activation, which is attenuated upon siRNA-mediated knockdown of either cGAS or STING. With a mechanism unique to KSHV, vIRF1 disrupts the TBK1-STING interaction by interacting with STING itself, thereby preventing STING activation [136]. This is a clever strategy that KSHV employs, as vIRF1’s temporal and overall expression is the earliest of the KSHV vIRFs [143]. Additionally, and perhaps not surprisingly, vIRF1, vIRF3, and vIRF4 were recently identified as components of the KSHV virion [144].

In addition to the antagonistic functions already described for vIRF1, this KSHV-specific protein also targets ISG15 in a multifaceted manner. vIRF1 directly interacts with cellular HERC5, the ISG15 E3 ligase, to inhibit ISG15. However, vIRF1 additionally interacts with ISG15 directly. In BCBL1 cells, which harbor KSHV latently, stably overexpressing vIRF1, attenuated ISGylation and IRF3 expression. Similarly, shRNA-mediated knockdown of ISG15 or HERC5 in iSLK.219 cells prior to the addition of reactivation stimuli resulted in a significant increase in KSHV lytic gene transcription and the production of infectious particles, compared to control cells [145]. Together, these findings suggest vIRF1 targets ISG15 to antagonize the type I interferon response, thereby allowing efficient KSHV replication.

### 4.3. Other KSHV Proteins That Regulate Host Innate Responses

Several TLRs are regulated following infection, across several different cell types. KSHV infection stimulates TLR3, an RNA sensor, in monocytes [146], while simultaneously activating TLR9, a DNA sensor, in plasmacytoid dendritic cells (pDCs) [147]. Activation of either of these TLRs results in a net upregulation of antiviral cytokines, coupled with the production of interferon. Despite this activation, the KSHV-encoded G protein-coupled receptor (vGPCR), encoded by *ORF74*, as well as vIRF1, discussed in detail above, downregulate TLR4 [148]. Additionally, *ORF50*-encoded RTA (discussed in detail in the next section) attenuates TLR3 and TRL4 signaling [149,150], suggesting KSHV had devised several strategies to target these PRRs.

KSHV ORF20 is a member of the UL24 herpesvirus gene family, thus is conserved across the three herpesvirus subfamilies. Largely uncharacterized, Bussey et al. recently showed KSHV ORF20 interacts with OAS-like protein (OASL) [151], an ISG that functions to inhibit the replication of a wide range of RNA viruses (reviewed in [152]). In addition to their interaction, ORF20 upregulates *OASL* gene expression in an IRF3-dependent, IFNAR-independent fashion [151]. However, unlike OASL’s role in inhibiting a variety of RNA viruses, this ISG appears to enhance KSHV infection [151], suggesting KSHV has evolved to utilize this ISG to its benefit rather than to its detriment. Both ORF20 and OASL are upregulated following viral reactivation [151], and thus perhaps KSHV coopted this otherwise host antiviral protein to ensure reactivation from latency, thereby priming the cell to bolster subsequent lytic replication.

KSHV *ORF10* encodes a protein termed, regulator of IFN function (RIF), based on the finding that its expression repressed IFN-induced ISRE-luciferase activity in an unbiased screen of 80 KSHV ORFs. By forming complexes that contain the kinases Jak1 and Tyk2, as well as STAT2, RIF abrogates IFN signaling, as the two kinases are inhibited and STAT2 activation is attenuated [153]. While recent findings suggest RIF is not incorporated into mature, viral particles [144], it is expressed soon after infection. Consistent with earlier findings [154], RIF was classified with delayed-early kinetics [153]. Thus, this mechanism by which KSHV antagonizes the innate response could act subsequent to those mediated by incoming tegument proteins. This strategy, similar to one HCMV uses, likely maintains some level of continued subversion of the host innate immune response as infection progresses.

### 4.4. Latency and Reactivation—Roles for RTA and LANA in Innate Immune Evasion

As mentioned above, KSHV’s manipulation of the innate immune response is not restricted to the lytic phase of the viral life cycle. KSHV latency and reactivation have also been studied in this context, with a particular focus on two viral-encoded proteins important to these stages of infection. *ORF50* encodes the immediate early replication and transcription activator (RTA) protein, which is a transcription factor that is also regarded as the “lytic switch” that facilitates reactivation from latency [155]. RTA, like the vIRFs and ORF45, targets IRF7 by exerting E3 ligase activity, thereby targeting IRF7 for proteasomal degradation [156]. RTA also promotes proteasomal degradation of TLR3 [150] and inhibits TLR4 signaling via degradation of *MYD88* transcripts [149]. These likely represent mechanisms KSHV has developed to antagonize innate responses to ensure successful viral reactivation into the lytic life cycle. ORF73-encoded LANA, as its name implies, is a robust, latently encoded protein that tethers the viral genome to host chromosomes via direct interaction with the viral terminal repeats. Additionally, LANA has a number of other functions, having roles in viral genome replication, epigenetic modification, viral promoter regulation, and cell signaling modulation, to name a few (reviewed in [157]). As such a multifunctional protein, it comes as no surprise that LANA also antagonizes host innate responses. For example, LANA binds the IFNβ promoter, thereby inhibiting *IFNB* transcription and effectively dampening the IFN response [158]. As mentioned above, LANA also binds and inhibits cGAS [137]. Collectively, these data suggest KSHV manipulates the host innate immune response even during latency and reactivation. While such strategies are likely required for the virus to successfully overcome these strong host defenses. In support of this, RIG-I/MAVS knockdown resulted in an increase in viral reactivation [120], suggesting subversion of this potent innate response is required for viral reactivation. It is attractive to speculate RIG-I/MAVS signaling impacts the reactivation of other herpesviruses in a similar fashion, though additional work in cell and animal systems specific to each herpesviral subfamily will be necessary to thoroughly parse these details. Alternatively, it is possible that some level of RIG-I-/MAVS signaling is required to maintain viral latency; perhaps a continual priming of the innate host response may function to maintain KSHV in its latent state. Further examination of host responses during various phases of infection will bolster our understanding of what is likely a very fine balance between the host and the virus.

## 5. Conclusions

The recognition of foreign pathogens initiates the host innate immune response, representing the first defense employed by a cell undergoing viral infection. For every newly identified antiviral measure, successful viruses efficiently undermine these responses. Arguably, human herpesviruses are the masters at derailing the innate immune pathway, as they have evolved a myriad of countermeasures to subvert antiviral defenses, thereby ensuring infection, propagation, and ultimately, survival within a population. Our understanding of these complex processes is far from complete. It is evident, however, that as we learn more about such host-pathogen interactions, we will begin to expose potential vulnerabilities, opening the door to novel targets for therapeutic interventions. The interplay between host and pathogen is a constant battle, but exploiting specific innate defenses could indeed shift from the viral advantage, ensuring a ‘win’ for the host. 

## Figures and Tables

**Table 1:** HSV-1-encoded proteins target the host innate response.

Herpesvirus	Viral Protein	Host Cell Target
**HSV-1**	ICP0	inhibits TLR2
		decreases TLR adaptor proteins (MyD88, TIRAP)
		degrades IFI16
	US3	inhibits TLR3 expression
		inhibits TRAF6
		hyperphosphorylates IRF3 to prevent TBK1-induced activation
		hyperphosphorylates p65 to prevent IFNβ induction
	US11	blocks RIG-I & MDA5 interaction with MAVS
		inhibits PKR
		abrogates IFN-induced OAS activation
	UL36	degrades TRAF3, preventing TBK1 recruitment & IRF3 activity
	UL37	blocks cGAS enzymatic activity
	ICP27	targets TBK1-mediated STING signaling
	UL41	binds STING & prevents its translocation to the ER
		blocks IFIT3 activity
		targets ISGs
	ICP34.5	sequesters TBK1 to prevent IRF3 activation
	UL46	inhibits TBK1
	VP16	blocks IRF3-CBP interaction
	Vhs	inhibits tetherin

**Table 2:** Multiple HCMV-encoded proteins usurp the innate immune response.

Herpesvirus	Viral Protein	Host Cell Target
**HCMV**	pp65	interacts with & blocks IFI16 signaling
		interacts with & blocks STING signaling
		binds & inactivates cGAS
	pUL94	interacts with & blocks STING signaling
	pp71	interacts with & blocks STING signaling
	pUL26	suppresses accumulation of ISGylated proteins via ISG15 interaction
	IE1	attenuates *ISG15* expression
	pUL50	inhibits global ISGylation
	pUL23	interacts with NMI, restricting ISG transcription via STAT1
		targets STING for degradation
	IE2	binds NFκB, abrogating its binding to the IFNβ promoter
	UL37x1/vMIA	suppresses MAVS signaling
		degrades RIG-I
	pUS9	interacts with STING, blocking IRF3 activity
		targets MAVS, preventing TBK1 & IRF3 recruitment
	pUL42	interacts with cGAS, blocking cGAMP production
		interacts with and retains STING in the ER, preventing its activation
	pUL31	interacts with cGAS, blocking cGAMP production
	IRS1, TRS1	target OAS/Rnase L signaling
	pORF94	inhibits OAS1 expression and function

**Table 3:** KSHV employs multiple strategies to escape the innate immune response.

Herpesvirus	Viral Protein	Host Cell Target
**KSHV**	ORF64	Attenuates IFN signaling via disruption of RIG-I ubiquitination
	ORF45	Suppresses IRF7 activity & prevents its nuclear accumulation
	ORF63	Inhibits NLRP1-depended functions, including degradation of IL-1β & IL-18
	ORF52	Binding cGAS & DNA to prevent signaling to STING and TBK1
	LANA	Interacts with cGAS, preventing TBK1-IRF3 activation
		Binds the IFNβ promoter to limit transcription
	vIRF1	Inhibits TLR3-mediated type I IFN induction
		Downregulates TLR4
		Disrupts TBK1’s interaction with STING to abrogate its activation
		Antagonizes the ISG15-mediated type I response
	vIRF2	Inhibits TLR3-mediated type I IFN induction
	vIRF3	Inhibits TLR3-mediated type I IFN induction
	vIRF4	Interacts with IRF7 to prevent downstream IFNα production
		Targets cellular IRF4
	vGPCR	Downregulates TLR4
	RTA	Attenuates TLR3 & TLR4
		Targets IRF7 for degradation
	ORF20	Interacts with OASL
		Upregulates OASL
	RIF	Abrogates IFN signaling via JAK-STAT regulation
	K5	Antagonizes tetherin
